# The spatial distribution pattern of Connexin26 expression in supporting cells and its role in outer hair cell survival

**DOI:** 10.1038/s41419-018-1238-x

**Published:** 2018-12-05

**Authors:** Sen Chen, Kai Xu, Le Xie, Hai-Yan Cao, Xia Wu, An-Na Du, Zu-Hong He, Xi Lin, Yu Sun, Wei-Jia Kong

**Affiliations:** 1 Department of Otorhinolaryngology, Union Hospital, Tongji Medical College, Huazhong University of Science and Technology, 430022 Wuhan, China; 2 Institute of Otorhinolaryngology, Tongji Medical College, Huazhong University of Science and Technology, 430022 Wuhan, China; 3 Department of Ultrasound, Union Hospital, Tongji Medical College, Huazhong University of Science and Technology, 430022 Wuhan, China; 4 Center of Instrumental Analysis and Metrology, Wuhan Institute of Virology, Chinese Academy of Sciences, 430071 Wuhan, China; 5 Department of Otolaryngology Head and Neck Surgery, Emory University School of Medicine, Atlanta, GA 30322 USA

## Abstract

Mutations in the *GJB2* gene (which encodes Connexin26 (Cx26)) account for about a quarter of all cases of non-syndromic deafness. Previous studies have indicated that knockout (KO) of *Gjb2* gene during early postnatal days can cause outer hair cell (OHC) loss in mouse models. However, the postnatal spatial distribution pattern of Cx26 in different types of supporting cells (SCs) and the role of such distributions for the survival of OHCs is still obscure. In this study, the spatial distribution patterns of Cx26 in SCs were observed, and based on these observations different spatial Cx26-null mouse models were established in order to determine the effect of changes in the spatial distribution of Cx26 in SCs on the survival of OHCs. At postnatal day (P)3, unlike the synchronous expression of Cx26 along both longitudinal and radial boundaries of most types of SCs, Cx26 expression was primarily observed along the longitudinal boundaries of rows of Deiter’s cells (DCs). From P5 to P7, radial expression of Cx26 was gradually observed between adjacent rows of DCs. When *Gjb2* gene was knocked out at random in different types of SCs, about 40% of the total DCs lost Cx26 expression and these Cx26-null DCs were distributed randomly in all three rows of DCs. The mice in this randomly Cx26-null group showed normal hearing and no significant OHC loss. When using a longitudinal KO pattern to induce knockout of *Gjb2* gene specifically in the third row of DCs, about 33% of the total DCs lost Cx26 expression in this specific longitudinally Cx26-null group. The mice in this group showed late-onset hearing loss and significant OHC loss, however, the morphology of corresponding DCs was slightly altered. In both experimental groups, no substantial DC loss was observed. These results indicate that longitudinal Cx26-based channels are predominant in DCs during P3–P5. The Cx26 expression along rows of DCs might play a key role in the survival of OHCs, but this longitudinal KO pattern in DCs has a limited effect on DC survival or on its postnatal development.

## Introduction

Mutations in the *GJB2* gene (which encodes Connexin26 (Cx26)) account for a quarter of all cases of non-syndromic deafness in different populations^1–3^. Cx26 self-assembles to form intercellular channels called gap junctions, and these allow ions, second messengers, microRNAs, and other small molecules to pass through^4–6^. Several different mouse models have been established to study the mechanism through which *GJB2* mutations lead to deafness, but this process is still poorly understood.

In the inner ear of mice and humans, the Cx26 protein is not expressed in auditory sensory hair cells (HCs), but it is expressed in different types of supporting cells (SCs) in the cochlear epithelium^7–9^. In the mouse cochlea, Cx26 is first detected in a few cells on embryonic day 14.5, and the region of cochlear Cx26 expression gradually expands during postnatal development^10^. Radial sections show that the Cx26 protein is found in the cells of the greater epithelial ridge (GER) at postnatal day (P)2, and pillar cells (PCs), Deiter’s cells (DCs), and Claudius cells (CCs) show punctate Cx26 signals at P3. By P7, Cx26 is expressed in all types of SCs in the cochlear epithelium^11^. These studies have provided the time-sequence expression pattern of cochlear Cx26 in the radial plane. However, the cochlear epithelium is a finely detailed spatial structure, these spatial distribution patterns of cochlear Cx26 during different stages of postnatal development still need further study.

According to previous studies, loss of outer HCs (OHCs) and developmental abnormalities in SCs were the two main pathological changes in different *Gjb2* mutation or knockout (KO) mouse models^12–18^. In these models, cochlear *Gjb2* gene could be extensively knocked out in different types of SCs, and rapid loss of SCs and OHCs was seen at P14–P15^17,18^. The changes in the *Gjb2* gene were induced at embryonic or early postnatal days in these models. When cochlear Cx26 was reduced in the mature cochlea (P16 or P18), the mice showed normal hearing with no significant OHC or SC loss over the course of at least 1 month^19,20^. These results indicated that early postnatal Cx26 expression might play a vital role in the survival of both OHCs and SCs. However, the specific mechanism by which early postnatal Cx26 expression affects the survival of cochlear cells is still obscure.

In this study, flattened cochlear preparations combined with radial sections were used to investigate the spatial distribution of Cx26 in different SCs at different postnatal time points. Furthermore, two distinct spatial Cx26-null mouse models were established to study the effect of the spatial distribution of Cx26 in SCs on the survival of OHCs. Use of the Lgr5 promoter allowed us to establish a specific longitudinally Cx26-null mouse model, and in these mice *Gjb2* gene was knocked out in all of the third row DCs (DC3s) and some of the inner PCs (IPCs). The second mouse line carried the Rosa26 promoter, and injection of a small dose of tamoxifen into these mice caused random cells in all three rows of DCs and other SCs to lose their Cx26 expression. Comparisons between these two models in terms of the relationship between the number of Cx26-null DCs in a row and the rate of OHC loss have provided a better understanding of the effect of the early postnatal Cx26 distribution patterns of SCs on the survival of OHCs.

## Results

### The distinct distribution patterns of Cx26 in different types of SCs during early postnatal development

**Fig. 1:**
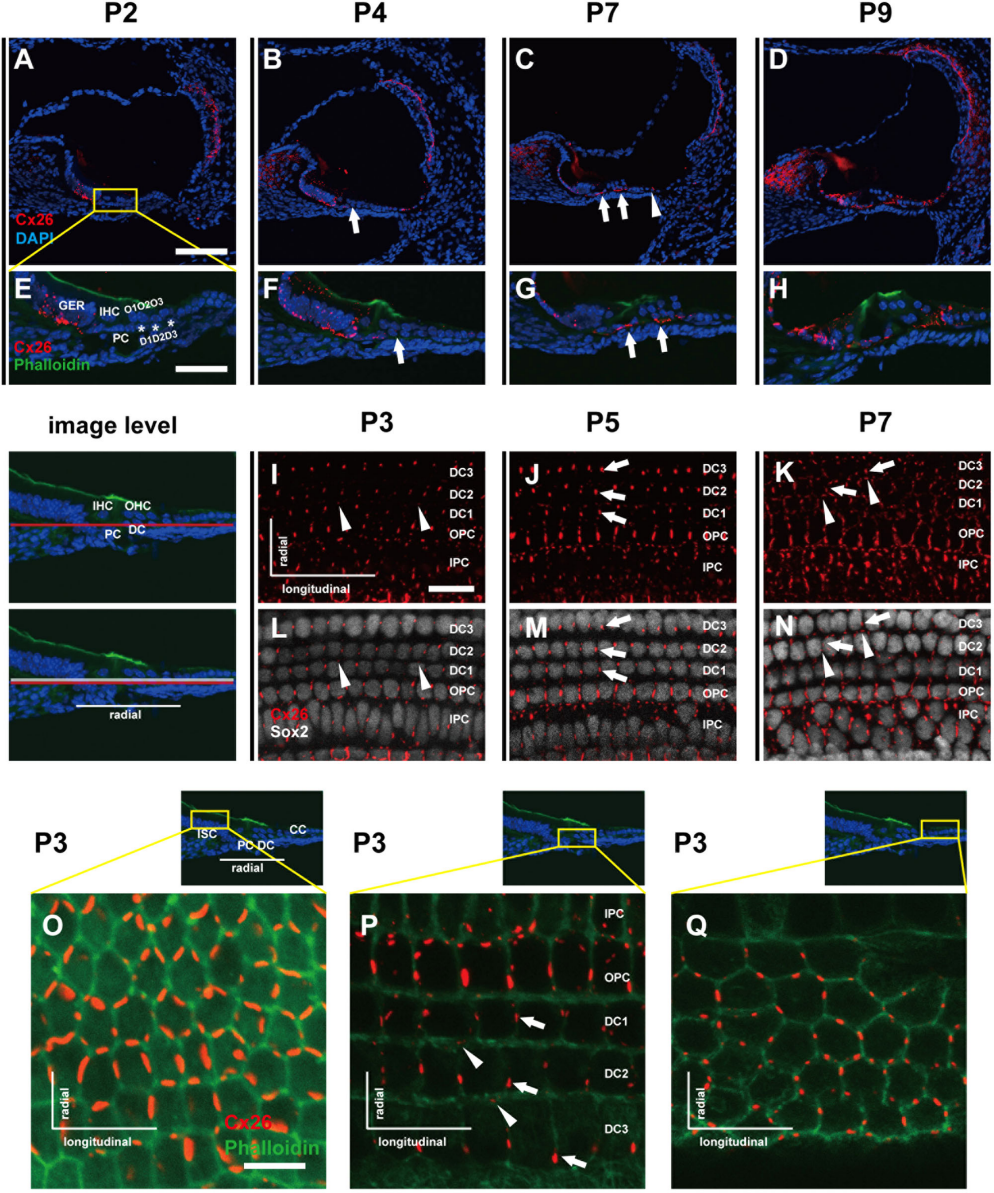
Distribution patterns of Cx26 in different SCs of the mouse cochlea at different postnatal time points. **a**–**d** Postnatal Cx26 immunolabeling (red) in cochlear radial sections. The yellow box in **a** indicates the location of the organ of Corti. **e**–**h** Postnatal Cx26 immunolabeling (red) in radial sections of the organ of Corti. Asterisks indicate the different rows of DCs. D1:DC1, the first row of DCs; O1:OHC1, the first row of OHCs. White arrows and arrowheads indicate different SCs that show Cx26 expression. **i**–**k** Postnatal Cx26 immunolabeling (red) in flattened preparations. **l**–**n** Postnatal Cx26 (red) and Sox2 (white) immunolabeling in corresponding flattened preparations. Images of the radial section (left) are given to show the levels of the images. The white arrowheads indicate Cx26 expression on the radial sides of DCs. The white arrows indicate longitudinal Cx26 expression in DCs. **o** Cx26 immunolabeling in ISCs at P3. **p** Cx26 immunolabeling in different rows of DCs at P3. **q** Cx26 immunolabeling in CCs at P3. Images of the radial section are shown on top of **o**, **p**, and **q**, and the yellow boxes in the images are the areas of magnification in **o**, **p**, and **q**. GER great epithelial ridge, ISC inner sulcus cell, PC pillar cell, IHC inner hair cell, OHC outer hair cell, CC Claudius cell. The scales in panels **a**, **e**, **i**, and **o** represent 100, 40, 30, and 10 µm, respectively

In cochlear radial sections, the Cx26 signal (red) was detected in cells of the GER at P2 (Figs. 1a, e). At P4, weak Cx26 signals could be detected in both PCs and DCs (white arrows, Figs. 1b, f). At P7, the Cx26 signals were found in greater numbers of SCs (white arrows and arrowhead, Figs. 1c, g). At P9, Cx26 signals were detected in almost all SCs but not in HCs (Figs. 1d, h). The flattened preparations provided a more detailed view of the spatial distribution of Cx26. At P3, the Cx26 staining (red) was clearly shown in DCs and PCs. Unlike PCs, which had both longitudinal and radial distributions of Cx26, the Cx26 in DCs was mainly expressed longitudinally in rows of DCs. At P3, a very small amount of Cx26 expression was observed at the boundary between two adjacent rows of DCs (radial expression, white arrowheads, Figs. 1i, l). Compared with the radial expression of Cx26 in DCs, the longitudinal expression of Cx26 in DCs was still predominant at P5 (white arrows, Figs. 1j, m). By P7, however, this longitudinally dominant Cx26 distribution pattern in DCs disappeared. At this time, the Cx26 signal was almost the same along both the longitudinal and radial boundaries of all DCs (Figs. 1k, n). To confirm this unique distribution, the distribution patterns of Cx26 in different SCs were compared at P3. The cell membranes were labeled by phalloidin (green). In inner sulcus cells (ISCs) and CCs, similar Cx26 signals were evenly distributed along all of the cells’ boundaries (Figs. 1o, q). However, a strong Cx26 signal (white arrows, Fig. 1p) was clearly observed longitudinally in the same row of DCs. Occasionally, weak Cx26 signals in DCs (white arrowheads, Fig. 1p) were detected at the boundaries between adjacent rows of DCs.

### Two distinct spatial Cx26-null mouse models were established

**Fig. 2:**
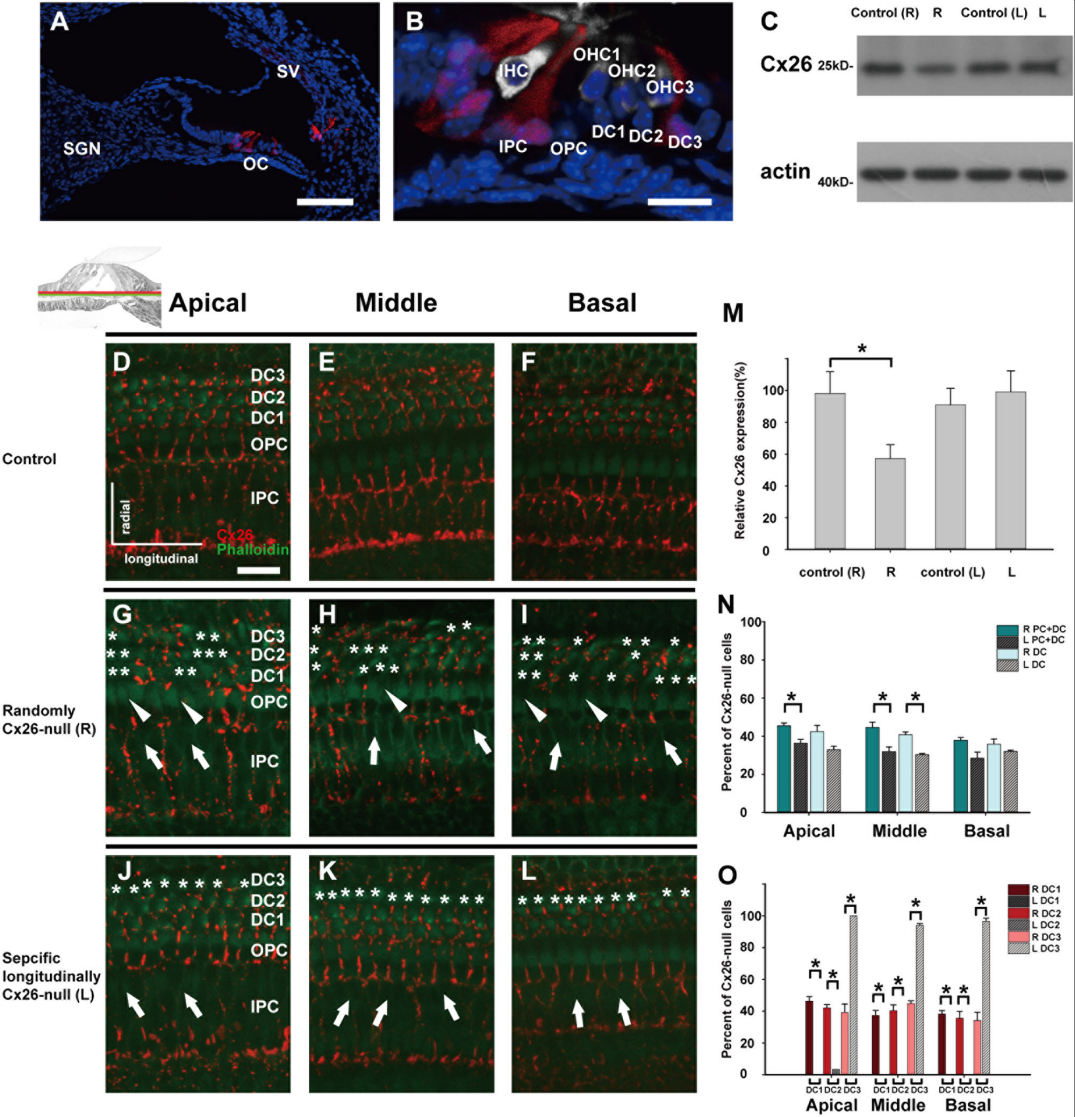
Cx26 expression patterns in different experimental groups. **a**, **b** The tdTomato staining (red) shows that Cre was activated in cells in the cochlea (**a**) and the organ of Corti (OC) (**b**). **c** Representative western blot results show Cx26 expression in the control groups, the randomly Cx26-null group (R group, R), and the specific longitudinally Cx26-null group (L group, L) at P7. **d**–**f** Cx26 immunolabeling in the apical, middle, and basal turns of the control group, respectively. **g**–**i** Cx26 immunolabeling in the apical, middle, and basal turns of the R group, respectively. **j**–**l** Cx26 immunolabeling in the apical, middle, and basal turns of the L group, respectively. Asterisks indicate the Cx26-null DCs. The white arrowheads indicate the region of Cx26-null OPCs, whereas the white arrows indicate the region of Cx26-null IPCs. **m** Relative expression of cochlear Cx26 at P7 (*n* = 4 in each group) in the control and experimental groups. **n** Cx26-null cell counts at P7 in different turns of the cochlea from different experimental groups (*n* = 3 in each group). **o** Cx26-null DC counts at P7 (*n* = 3 in each group). *Significant difference between two groups (*P* < 0.05). SV striae vascularis, SGN spiral ganglion neuron. The scales in panels **a**, **b**, and **d** represent 100, 30, and 30 µm, respectively

To confirm the spatial KO patterns of transgenic mice upon activation of the Lgr5 promoter, the expression of Cre was visualized by tdTomato. Consistent with a previous report, the tdTomato signals (red) were mainly observed in DC3s, IPCs, and inner phalangeal cells at P7 (Figs. 2a, b)^21^. Next, the expression levels of cochlear Cx26 were evaluated at P7. The littermates without Cre were used as controls, therefore two control groups (one for the randomly Cx26-null group (control R) and one for the specific longitudinally Cx26-null group (control L)) were included in the statistics. Western blot quantification showed that cochlear Cx26 expression in the randomly Cx26-null group decreased to 57.2 ± 8.8% (*n* = 4, *P* < 0.05). However, there was no statistically significant difference between the control L and specific longitudinally Cx26-null groups (*n* = 4, *P* = 0.32). To better understand the residual Cx26 expression in these models, Cx26-null cells were quantified at P7. In the randomly Cx26-null group, the Cx26-null cells were observed in nearly all types of SCs. Cx26-null DCs (asterisks in Figs. 2g–i) were observed in different rows (DC1, DC2, and DC3), and a number of IPCs (white arrows, Figs. 2g–i) and OPCs (arrowheads, Figs. 2g–i) lost Cx26 expression in this group. However, in the specific longitudinally Cx26-null group, nearly all DC3s (asterisks, Figs. 2j–l) lost their Cx26 signal, whereas the DC1s and DC2s still had normal Cx26 expression. In this group, all of the OPCs still had Cx26 expression, whereas some IPCs lost Cx26 expression (white arrows, Figs. 2j–l). In the randomly Cx26-null group (*n* = 3), quantitative analysis showed that the proportions of Cx26-null SCs (DCs and PCs) were 45.5 ± 1.4%, 44.7 ± 2.8%, and 37.9 ± 1.4% in the apical, middle, and basal turns of the cochlea, respectively. However, the proportions of Cx26-null SCs (DCs and PCs) in the specific longitudinally Cx26-null group (*n* = 3) were 36.3 ± 2.1%, 31.9 ± 2.5%, and 28.6 ± 3.1% in the apical, middle, and basal turns, respectively. Additionally, the proportions of total Cx26-null DCs in the randomly Cx26-null group (*n* = 3) were 42.5 ± 3.2%, 40.8 ± 1.6%, and 36.0 ± 2.6% in the apical, middle, and basal turns, respectively. However, the proportions of total Cx26-null DCs in the specific longitudinally Cx26-null group (*n* = 3) were 33.0 ± 1.8%, 30.5 ± 0.5%, and 32.0 ± 0.7% in the different turns, respectively (Fig. 2n). Furthermore, Cx26-null DCs in the different rows were also quantified. In the specific longitudinally Cx26-null group, only DC3s lost their Cx26 expression (>95%). However, about 40% of all DC1s, DC2s, and DC3s lost their Cx26 expression in the randomly Cx26-null group (Fig. 2o).

### Late-onset high-frequency hearing loss was seen in the specific longitudinally Cx26-null group

**Fig. 3:**
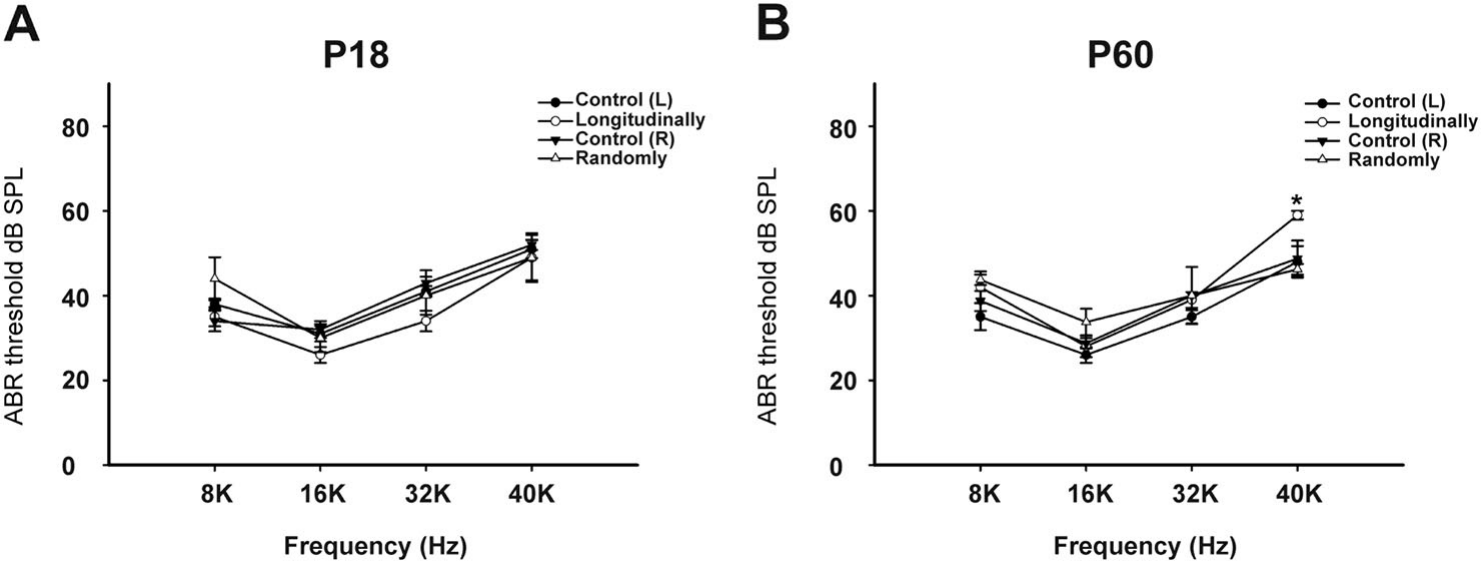
Late-onset high-frequency hearing loss was observed in the longitudinally Cx26-null group. **a**, **b** ABRs were measured in different control and experiment groups at P18 (**a**) or P60 (**b**). *Significantly different from the control group (*P* < 0.05). Longitudinally: specific longitudinally Cx26-null group; Randomly: the randomly Cx26-null group

The auditory brainstem response (ABR) was tested at P18 and P60. The littermates without Cre were tested as controls; therefore, two control groups (control R and control L) were included in the statistics. At P18, all four groups of mice (*n* = 5 in each group) showed normal hearing. At P60, the hearing thresholds of the specific longitudinally Cx26-null group at 8, 16, 32, and 40 kHz were 42.0 ± 3.7, 28.0 ± 2.5, 39.0 ± 1.9, and 59.0 ± 1.0 dB SPL, respectively, and the hearing thresholds of the corresponding control group at 8–40 kHz were 35.0 ± 3.2, 26.0 ± 1.9, 35.0 ± 1.6, and 48.0 ± 3.7 dB SPL, respectively. Compared with controls, there was a statistically significant difference at 40 kHz (*n* = 5, *P* = 0.02) (Fig. 3).

### OHC loss without corresponding DC loss was observed in the basal turn of the cochlea from the specific longitudinally Cx26-null group

**Fig. 4:**
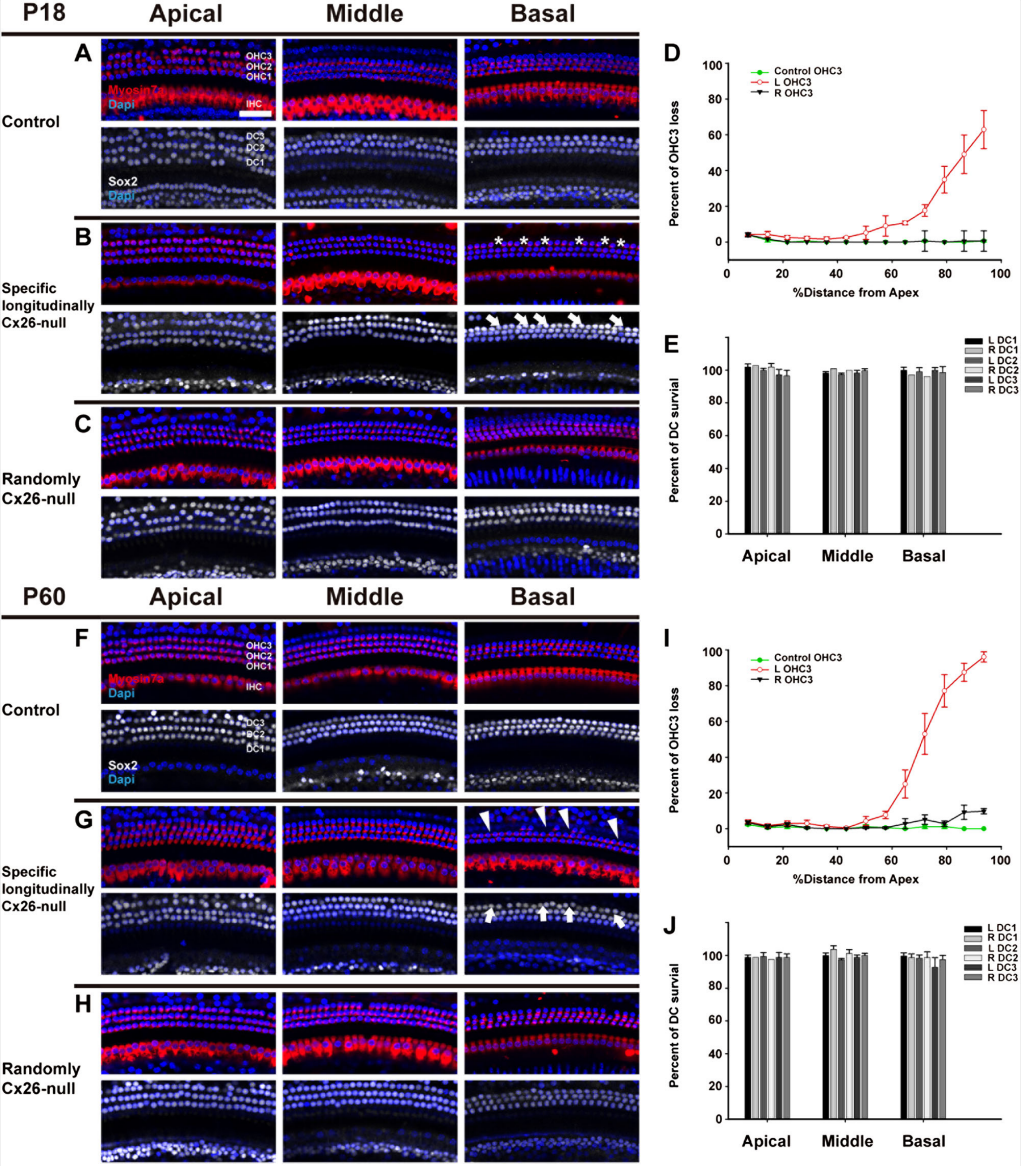
OHC and DC counts in the control and experimental groups. **a**–**c** Representative images of HCs (Myosin7a, red) and corresponding DCs (Sox2, white) of different turns in control (**a**), specific longitudinally Cx26-null (**b**), or randomly Cx26-null groups (**c**) at P18. Asterisks indicate the missing OHC3s (**b**), and white arrows indicate the corresponding DC3s (**b**). **d** Quantifications of OHC3 loss at specific cochlear locations in the different groups at P18. **e** Quantifications of DC’s survival at specific cochlear locations in the different groups at P18. **f**–**h** Representative images of HCs (Myosin7a, red) and corresponding DCs (Sox2, white) of different turns in control (**f**), specific longitudinally Cx26-null (**g**), or randomly Cx26-null groups (**h**) at P60. White arrowheads indicate the regions of missing OHC3s (**g**), and white arrows indicate the regions of corresponding DC3s in specific longitudinally Cx26-null group at P60 (**g**). **i** Quantifications of OHC3 loss at specific cochlear locations in the different groups at P60. **j** Quantifications of DC’s survival at specific cochlear locations in the different groups at P60. The scales in panel **a** represent 40 µm

Quantification of OHCs and DCs was performed at P18 and P60. The mice from the two control groups showed no differences in the ABR test, so the two control groups were combined into one group for quantification analysis (*n* = 4). In the randomly Cx26-null group, there was no obvious OHC or DC loss at P18 (Fig. 4c). However, in the specific longitudinally Cx26-null group, scattered OHC3 (the third row of OHC) loss was found in the basal turn (asterisks, Fig. 4b), whereas their corresponding DCs were still intact at P18 (white arrows, Fig. 4b). Additionally, a cochleogram showed that 17.7 ± 3.3%–62.9 ± 10.7% of OHC3 loss was seen in the cochlear middle to basal turn of the specific longitudinally Cx26-null group at P18 (Fig. 4d). No substantial DC loss was observed in either of the two experimental groups at P18 (Fig. 4e).

At P60, OHC3 loss was increased (white arrowheads, Fig. 4g) in the basal turn of the cochlea of the specific longitudinally Cx26-null group. Accordingly, no substantial DC loss was observed in the corresponding region (white arrows, Fig. 4g). Occasionally, a few missing OHCs were observed in the basal turn of the randomly Cx26-null group, whereas no obvious DC loss was found in the corresponding region (Fig. 4h). The cochleogram showed that 53.0 ± 11.4% to 96.1 ± 2.8% OHC3 loss was found from the middle to the basal turn of the cochlea of the specific longitudinally Cx26-null group at P60 (Fig. 4i). No substantial DC loss was observed in either of the two experimental groups at P60 (Fig. 4j). In two experimental groups, quantification analysis showed that no substantial OHC1 or OHC2 loss was found at P18 or P60 (Fig. S2 A-D).

**Fig. 5:**
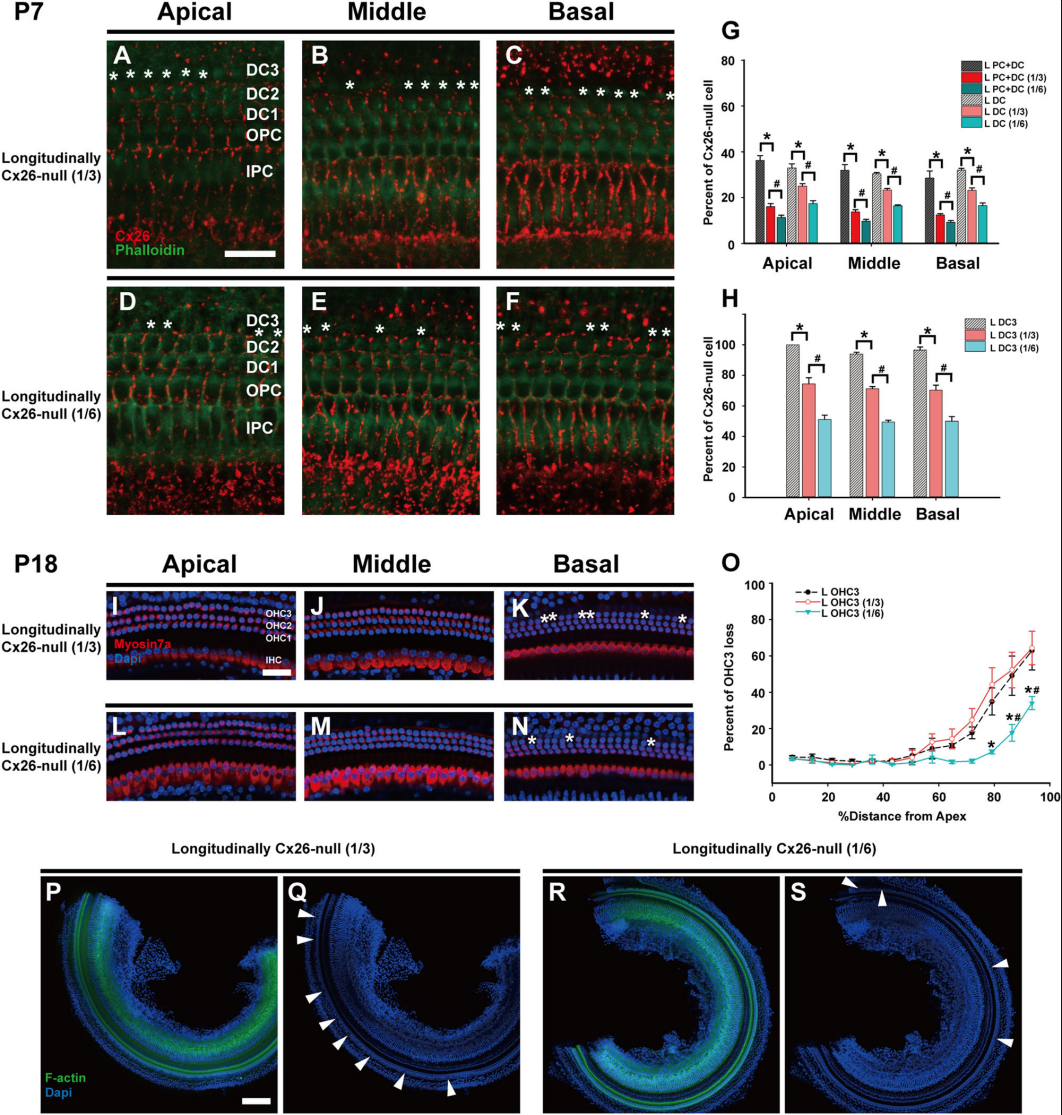
Cx26 expression patterns and OHC loss patterns in different longitudinally Cx26-null groups. **a**–**c** Cx26 immunolabeling (red) in the apical, middle, and basal turns of the longitudinally Cx26-null group (1/3), respectively. **d**–**f** Cx26 immunolabeling in the apical, middle, and basal turns of the longitudinally Cx26-null group (1/6), respectively. Asterisks indicate Cx26-null DCs. **g** Cx26-null cell counts at P7 in different turns of the organ of Corti (*n* = 3 in each group). **h** Cx26-null DC3 counts at P7 (*n* = 3 in each group). * and # indicate significant differences between two groups (*P* < 0.05). **i**–**k** Representative images of HCs in the apical (**i**), middle (**j**), and basal turns (**k**) from the longitudinally Cx26-null group (1/3). **l**–**n** Representative images of HCs in the different turns from the longitudinally Cx26-null group (1/6). **o** Quantifications of OHC3 loss at specific cochlear locations in different groups at P18. Asterisk indicates a significant difference compared with the specific longitudinally Cx26-null group. The hash symbol indicates a significant difference compared with the longitudinally Cx26-null group (1/3). **p**–**s** Representative images of the middle-basal parts of flattened cochlear preparations in different groups. White arrowheads indicated the regions of OHC3 loss. The scales in panels **a**, **i**, and **p** represent 30, 40, and 200 µm, respectively

Two other specific longitudinally Cx26-null mouse models were established by varying the dose of tamoxifen injection. In these two models, one-third or one-sixth of the standard tamoxifen dose was given in the same manner as in the specific longitudinally Cx26-null mouse lines and are referred to as the longitudinally Cx26-null group (1/3) and longitudinally Cx26-null group (1/6), respectively. In the longitudinally Cx26-null group (1/3) (*n* = 3), quantitative analysis showed that the proportions of SCs (DCs and PCs) without Cx26 were 16.1 ± 1.3%, 13.8 ± 0.9%, and 12.5 ± 0.6% in the apical, middle, and basal turns, respectively (Fig. 5g), and there were very few Cx26-null IPCs. In the longitudinally Cx26-null group (1/6) (*n* = 3), these proportions were reduced to 11.4 ± 1.0%, 9.7 ± 0.8%, and 9.2 ± 0.8% in the three turns of the cochlea, respectively (Fig. 5g). The proportions of Cx26-null DC3s were 74.5 ± 3.9%, 71.2 ± 1.4%, and 70.3 ± 3.3% in the different turns from the longitudinally Cx26-null group (1/3) (Fig. 5h), whereas they were 51.1 ± 2.7%, 49.4% ± 1.2%, and 49.9 ± 3.0% in the longitudinally Cx26-null group (1/6) (Fig. 5h). The cochleogram (*n* = 3 or 4) showed that the rates of OHC3 loss between the specific longitudinally Cx26-null group and the longitudinally Cx26-null group (1/3) were not significantly different. However, the OHC3 loss was significantly reduced in the longitudinally Cx26-null group (1/6) (Fig. 5o).

### Normal structure of the organ of Corti and no spiral ganglion neuron loss were observed in the specific longitudinally Cx26-null group

**Fig. 6:**
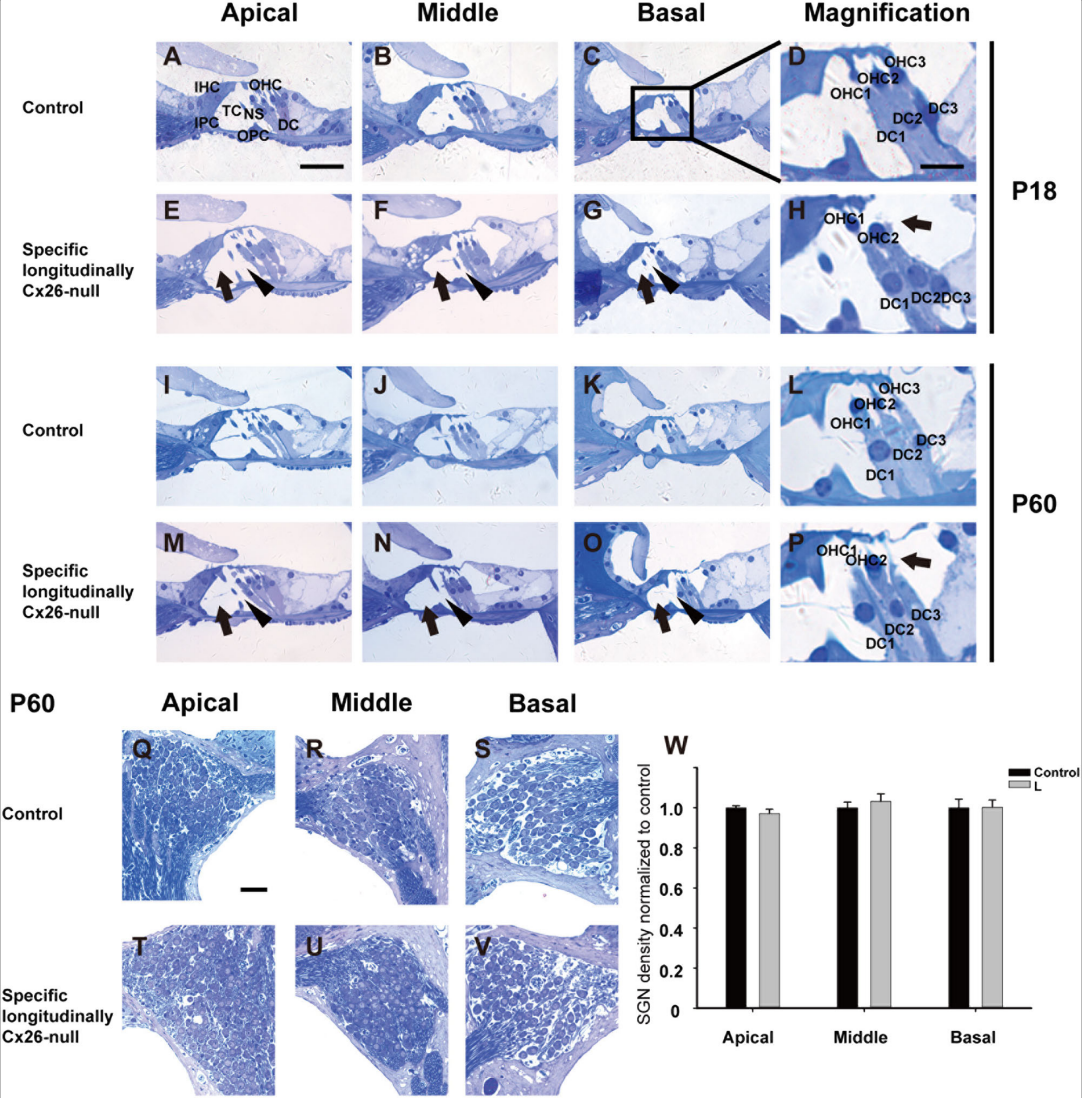
Cochlear morphology and spiral ganglion neuron counts in the specific longitudinally Cx26-null group. **a**–**c** The morphology of the organ of Corti in the apical (**a**), middle (**b**), and basal turns (**c**) of the control group at P18. **e**–**g** The morphology of the organ of Corti in different turns from the specific longitudinally Cx26-null group (the L group, L) at P18. The black arrows indicate the tunnel of Corti (TC), and the black arrowheads indicate Nuel’s space (NS). **d**, **h**, **l**, **p** Magnifications of the basal turn of the organ of Corti. The black arrows in panels **h** and **p** indicate the missing OHC3s. **i**–**k** The morphology of the organ of Corti in different turns from the control group at P60. **m**–**o** The morphology of the organ of Corti in different turns from the L group at P60. **q**–**s** The examples of the spiral ganglion neuron in the apical (**q**), middle (**r**), and basal turns (**s**) from control group at P60. **t**–**v** Examples of the spiral ganglion neuron in different turns from the L group at P60. **w** Spiral ganglion neuron counts in different groups at P60. The scales in panels **a**, **d**, and **q** represent 40, 20, and 30 µm, respectively

Radial sections showed that the tunnel of Corti was open and Nuel’s space was well formed at P18 in the specific longitudinally Cx26-null group (black arrows and arrowheads, Figs. 6e–g). Consistent with the HC counts, the OHC3s were lost in the basal turn (black arrow, Fig. 6h), whereas the DCs remained intact (Fig. 6h). The pathological change of the longitudinally Cx26-null group at P60 was similar to that at P18 (Figs. 6m–o), and the missing OHC3 can be also observed in the basal turn (black arrow, Fig. 6p). Additionally, there was no substantial spiral ganglion neuron loss in this group at P60 (Fig. 6w).

### The morphology of the phalangeal processes and the ultrastructure of DC3s in the specific longitudinally Cx26-null group

**Fig. 7:**
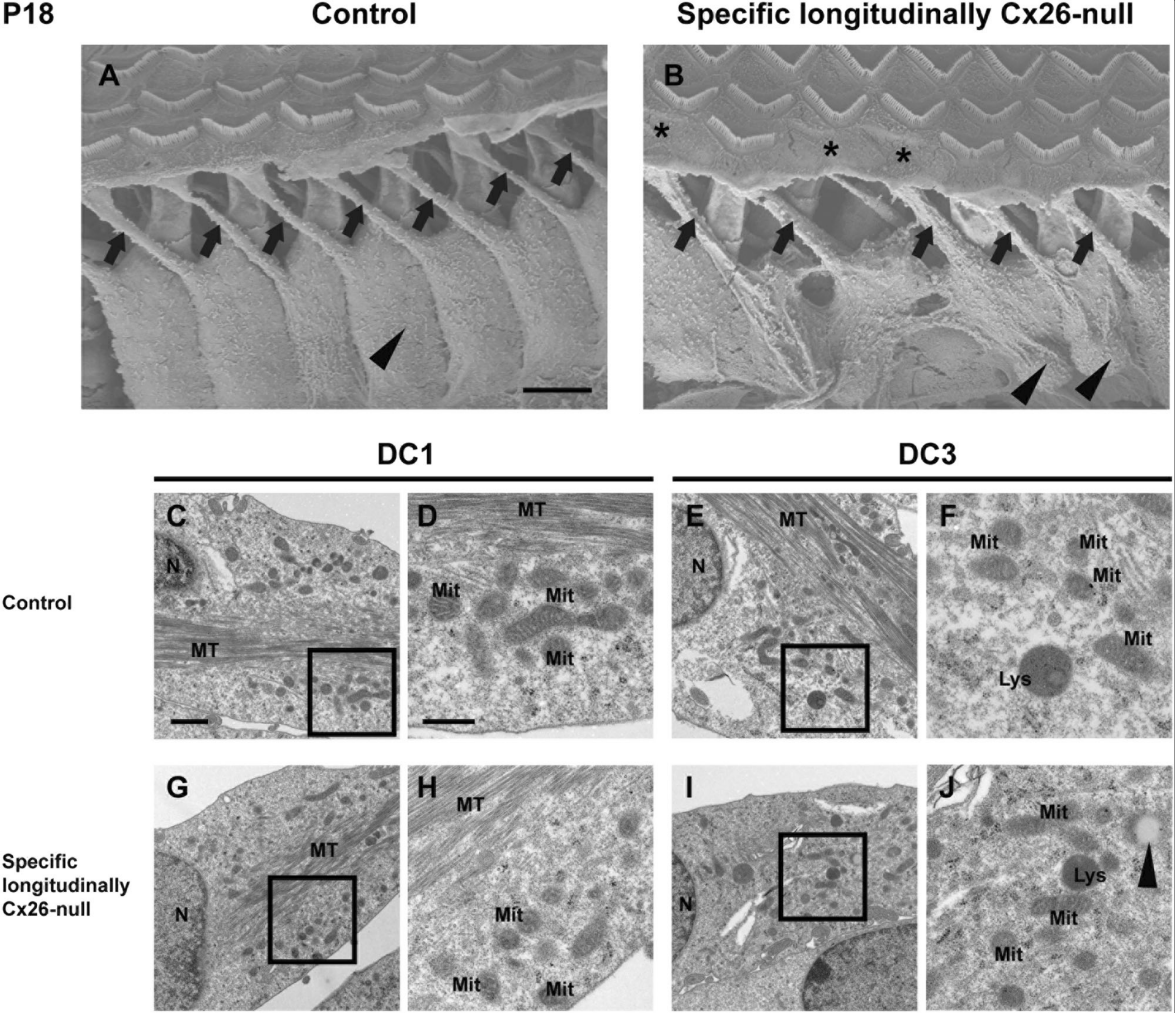
The ultrastructural changes in DCs from the specific longitudinally Cx26-null group. **a**, **b** The phalangeal processes and bodies of DC3s in basal turn were observed by scanning electron microscopy in the control (**a**) and the specific longitudinally Cx26-null group (**b**) at P18. The black arrows indicate the phalangeal processes, and the black arrowheads indicate the bodies of DC3s. Asterisks indicate the locations of missing OHC3s. **c**, **g** The ultrastructural morphology of DC1s in basal turn was observed by transmission electron microscopy in the control (**c**) and the specific longitudinally Cx26-null group (**g**) at P18. The black boxes in the left panels show the magnified area for organelle observation in the right panels. **e**, **i** The ultrastructural morphology of DC3s in basal turn was observed in the control (**e**) and the specific longitudinally Cx26-null group (**i**) at P18. The black arrowhead (**j**) indicates a lipid droplet. N nucleus, MT microtubule, Mit mitochondria, Lys lysosome. The scales in panels **a**, **c**, and **d** represent 5 µm, 1 µm, and 500 nm, respectively

In the control group, the phalangeal processes of DCs were thin cylindrical prominences (black arrows, Fig. 7a), and the bodies of the DCs (black arrowheads, Fig. 7a) were relatively uniform. In the specific longitudinally Cx26-null group, the phalangeal processes (black arrows, Fig. 7b) of the DC3s can develop into finger-like structures, but the diameters of the phalangeal processes changed a little bit, and most of them became larger. The bodies of the DC3s were not uniform, and some of the DC3 bodies were swollen (black arrowheads, Fig. 7b). The ultrastructural observation showed that DC1s in the specific longitudinally Cx26-null group were not affected, and bundles of microtubules and normal mitochondria (Figs. 7g, h) were observed in the bodies of DC1s. In DC3s, some lysosomes and mitochondria could be found in the perinuclear zone. Except for a few lipid droplets (black arrowhead, Fig. 7j), no signs of karyopyknosis, karyorrhexis, or autophagy were observed (Fig. 7i) in the DC3s from the specific longitudinally Cx26-null group.

## Discussion

Cx26 protein expression in DCs has both time-dependent and spatial distribution patterns. First, Cx26 is expressed longitudinally to form a dotted line pattern along each row of DCs at P3–P5. However, very little Cx26 is expressed at the boundaries between two adjacent rows of DCs during this period. At P5–P7, Cx26 begins to be expressed at the boundaries between two adjacent rows of DCs. These results suggest that intercellular communication within rows of DCs is preferentially built up at P3, and these longitudinal Cx26-based channels in rows of DCs predominate from P3 to P5. Previous studies showed the time course of Cx26 expression in radial sections, and Cx26 expression was initially observed in the cells of the GER and spread toward the cells in the lesser epithelial ridge (LER) from P1 to P8^10,11^. Thus, it would seem that the radial Cx26-based channels were established from the GER to the LER in turn, and these junctions are likely to be necessary for the radial delivery of small molecules between the GER and the LER. However, according to our observations, this radial intercellular communication between SCs might have limited effects in DCs during P3 to P5. Due to the lack of radial Cx26 expression in DCs, small molecules from the GER might be slowed down or even blocked in different rows of DCs during this period. In contrast, longitudinal delivery of small molecules through Cx26-based channels predominates within rows of DC at this time.

The postnatal expression of Cx26 in the organ of Corti is important for the survival of OHCs, and early postnatal Cx26 expression in rows of DCs might play a key role for the survival of OHCs. In the randomly Cx26-null group, numerous OPCs, IPCs, CCs, and ISCs had no Cx26 expression (Fig. S1). In the whole cochlea, Cx26 expression was reduced by about 42.8% in the randomly Cx26-null group, whereas there was no reduction in overall cochlear expression of Cx26 in the specific longitudinally Cx26-null group. However, OHC counts showed that no substantial OHC loss occurred in the randomly Cx26-null group. Thus, it would appear that KO of Cx26 in a certain number of SCs (except DCs) might not cause significant OHC loss. To validate this speculation, ISC-, IPC-, and CC-specific KO mouse models would be needed for further investigations.

The total proportion of Cx26-null DCs in the randomly Cx26-null group was about the same as that in the specific longitudinally Cx26-null group; however, OHC loss was only seen in the latter group. This observation indicated that there was no strong correlation between the total proportion of Cx26-negative DCs and OHC loss. However, the proportion of Cx26-null DC3s in the randomly Cx26-null group was about 40% (ranging from 36.0 ± 2.6% to 42.5 ± 3.2% in the different turns). By using different doses of tamoxifen injection (including 1/6, 1/3, or the full standard dose), these proportions could be manipulated to range from 49.4% to 100% in different longitudinally Cx26-null groups. In accordance with the rates of OHC loss in these groups, a strong correlation between the proportion of Cx26-negative DCs in a row and the corresponding OHC loss was seen. Based on our data, there might not be significant OHC loss when <40% of the DCs in a row lose Cx26 expression. OHC loss was observed in the basal turn when the proportion of Cx26-null DCs in a row exceeded 50%, and OHC loss reached its maximum when this proportion exceeded 70%.

Based on the above results, we propose the following hypothesis. From P3 to P5, the cells in rows of DCs are connected together by longitudinal Cx26-based channels. In other words, a row of DCs is connected by Cx26-related channels like a functional syncytium, which has a certain similarity with cardiomyocytes coupled by gap junctions. If >70% of the DCs lose Cx26 expression, this will disrupt the longitudinal intercellular communication in a row of DCs and severely impair the function of that row of DCs. This will ultimately lead to a significant loss of corresponding OHCs. When the proportion of Cx26-null DCs in a row is <40%, these residual Cx26-based channels might be sufficient to maintain the normal function of DCs and prevent the loss of OHCs. Another considerable factor is that we still lack the specific promoter for DC1 (or DC2), the accurate proportion of Cx26-null DC1 (or DC2) to maintain the survival of corresponding OHC1 (or OHC2) still needed further investigation.

Compared with the effect seen on OHCs, the two spatial Cx26-null patterns had no obvious negative effects on the survival of DCs or SGNs. In a previous study using a systemic Cx26-null mouse model, we found that the phalangeal processes of the three rows of DCs did not develop into finger-like structures^16^. Although the morphology of DC3s in the present work changed a little bit, the phalangeal processes of DC3s could still develop into finger-like structures in the specific longitudinally Cx26-null group. Moreover, the ultrastructure of DC3s showed no obvious pathological changes in this group. These results suggest that the longitudinal KO pattern in DCs has limited effect on DC’s survival or DC’s postnatal development.

The late-onset hearing loss at high frequency might be caused by OHC3 death in the basal turn in the specific longitudinally Cx26-null group. According to Muller’s formula, the frequency of 40 kHz corresponds to 75% distance from the apex^22^. At P18, the rate of OHC3 loss at the location near the 75% distance from the apex only ranged from 14.2 ± 3.3% to 33.2 ± 7.8%. If calculated by the total number of OHCs, the rate of OHC loss would not exceed 12%. This slight OHC loss might not cause significant hearing loss that can be detected by the ABR test. However, the rate of OHC3 loss near this location (for 40 kHz) ranged from 53.0 ± 11.2% to 77.2 ± 9.1% at P60, whereas the rate of OHC3 loss near the location responsible for hearing at 32 kHz was 25.0 ± 7.9% at this time point. Thus, it is reasonable that a statistically significant increase in hearing threshold was only found at 40 kHz.

**Fig. 8:**
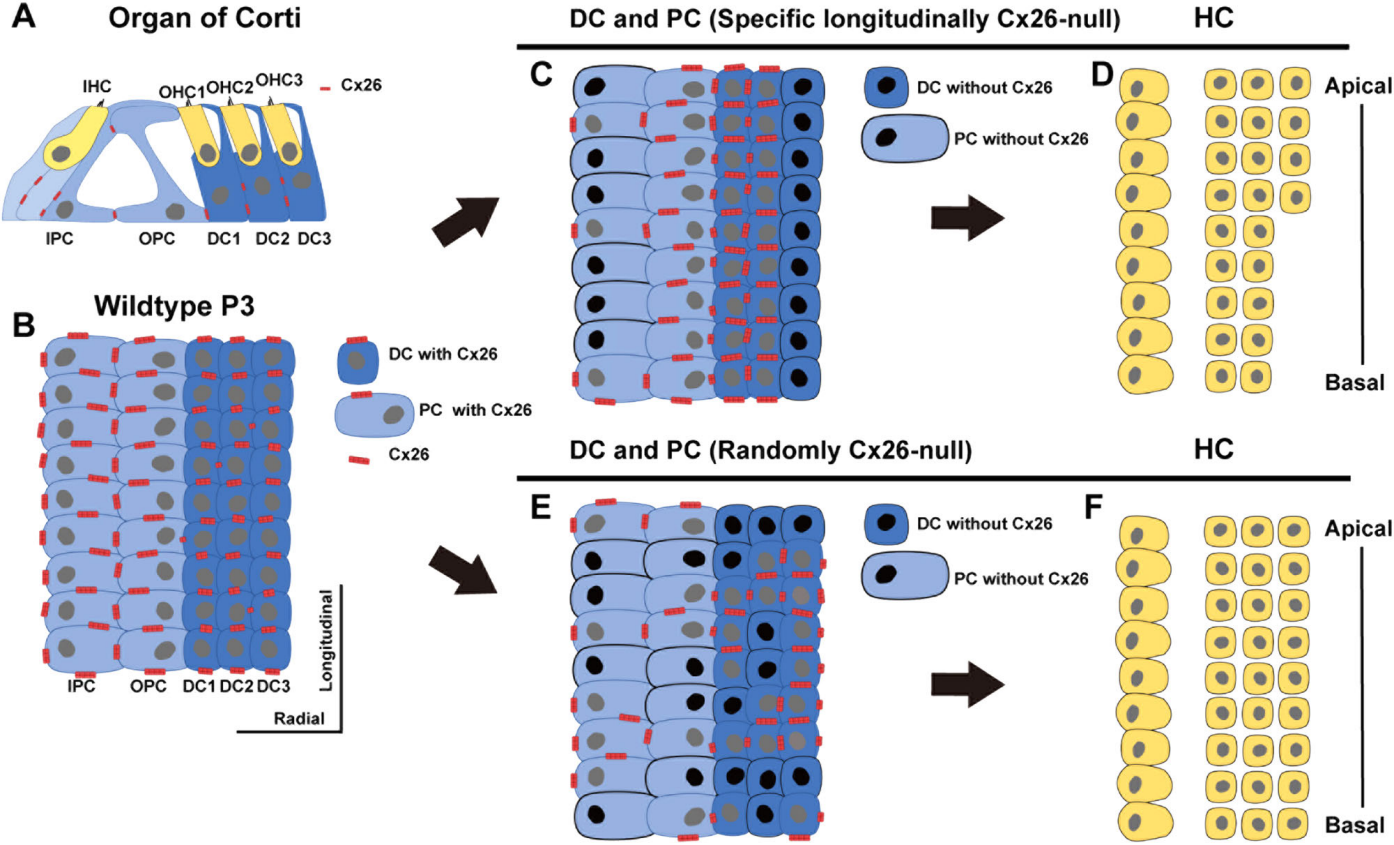
A cartoon showing the different spatial-specific Cx26-null patterns. **a** Cx26 expression (red) in the radial section of a mature organ of Corti. **b** Cx26 expression (red) in flattened preparations at P3. **c,** **e** Cx26 expression in the specific longitudinally Cx26-null (**c**) and randomly Cx26-null (**e**) groups at P7. **d**, **f** HC loss pattern in the specific longitudinally Cx26-null (**d**) and randomly Cx26-null (**f**) groups at P18

In conclusion, Cx26 expression in DCs has time-dependent and spatial distribution patterns during postnatal development. Cx26 is predominantly expressed longitudinally in rows of DCs during P3–P5 (Figs. 8a, b). In randomly Cx26-null group, about 40% of the total DCs lost Cx26 expression and these Cx26-null DCs were distributed randomly in all three rows of DCs (Fig. 8e). It would not cause significant OHC loss in this group (Fig. 8f). However, when using a longitudinal KO pattern to induce KO of *Gjb2* gene specifically in the third row of DCs (Fig. 8c), it can induce a substantial OHC3 loss in the basal turn (Fig. 8d). Our results indicated that the postnatal expression of Cx26 in the organ of Corti is important for the survival of OHCs, and this longitudinal expression of Cx26 in DCs during early postnatal development might play a key role in the survival of OHCs.

## Materials and methods

### Mouse models

Cx26^loxP/loxP^ mice and Rosa26CreER mice were provided by Prof. Xi Lin at Emory University. Lgr5^EGFP-Ires-CreERT2^ (Lgr5CreER) and ROSA26^CAG-loxP-stop-loxP^-tdTomato (tdTomato) mice were provided from Prof. Renjie Chai at Southeast University. Tamoxifen-inducible Cx26^loxP/loxP^;Rosa26CreER and Cx26^loxP/loxP^; Lgr5CreER mice were generated by crossbreeding of the Cx26^loxP/loxP^ mice with Rosa26CreER or Lgr5CreERmice, respectively. Mouse genotyping was performed by PCR amplification of tail genomic DNA. Details of the mice are given in our previous papers^15,19^. The genotyping primers were as follows:

Cx26 (F): 5ʹ-ACAGAAATGTGTTGGTGATGG-3ʹ,

Cx26 (R): 5ʹ-CTTTCCAATGCTGGTGGAGTG-3ʹ,

Rosa26Cre F): 5ʹ-AGCTAAACATGCTTCATCGTCGGTC-3ʹ,

Rosa26Cre (R): 5ʹ-TATCCAGGTTACGGATATAGTTCATG-3ʹ,

Lgr5-CreER (F): 5ʹ-CTGCTCTCTGCTCCCAGTCT-3ʹ,

Lgr5-wild-type (R): 5ʹ-ATACCCCATCCCTTTTGAGC-3ʹ,

Lgr5-mutant (R): 5ʹ-GAACTTCAGGGTCAGCTTGC-3ʹ,

tdTomato-wild-type (F): 5ʹ-AAGGGAGCTGCAGTGGAGT-3ʹ,

tdTomato-wild-type (R): 5ʹ-CCGAAA ATCTGTGGGAAGTC-3ʹ,

tdTomato-mutant (F): 5ʹ-GGCATTAAAGCAGCGTATC-3ʹ,

tdTomato-mutant (R): 5ʹ-CTGTTCCTGTACGGCATGG-3ʹ.

To confirm the activation of the Cre recombinase, we crossed Lgr5CreER mice with tdTomato mice, and tamoxifen (T5648-1G, Sigma-Aldrich, USA) was injected subcutaneously at P0 and P1 (the total dose was 1.5 mg/10 g body weight, once a day for 2 consecutive days). It was previously reported that CreER activation is tamoxifen dose-dependent;^23,24^ therefore, a smaller dose of tamoxifen (total dose 0.75 mg/10 g for 2 days) was administered to Cx26^loxP/loxP^; Rosa26CreER mice at P0 and P1, and cochlear Cx26 was partly and randomly knocked out in different types of cells of this group (the randomly Cx26-null group). To obtain the specific longitudinally Cx26-null mouse model, Cx26^loxP/loxP^; Lgr5CreER mice were given a tamoxifen dose of 1.5 mg/10 g for 2 days. Two reduced doses (total doses of 0.5 mg/10 g or 0.25 mg/10 g for 2 days) were injected into Cx26^loxP/loxP^; Lgr5CreER mice to establish the specific longitudinally Cx26-null group (1/3) and the specific longitudinally Cx26-null group (1/6), respectively. The littermates without Cre were used as controls.

All mice were raised in the specific-pathogen free Experimental Animal Center of Huazhong University of Science and Technology. All experimental procedures were conducted in accordance with the policies of the Committee on Animal Research of Tongji Medical College, Huazhong University of Science and Technology.

### ABR

ABR was performed at P18 and P60. As we previously reported, mice (*n* = 6–8 in each group) were anesthetized by intraperitoneal injection with a mixture of ketamine (120 mg/kg i.p.) and chlorpromazine (20 mg/kg, i.p.). Body temperature was maintained by placing the anesthetized mice on a heating pad. The recording electrode was placed at the vertex of the skull, and the reference electrode was inserted at the tested ear with a ground electrode at the contralateral ear. Tone bursts of 8, 16, 32, and 40 kHz were generated and responses were recorded using a Tucker-Davis Technologies System (RZ6, Tucker-Davis Tech., USA). The responses were the average from 1024 stimuli and were recorded in decreasing 10 dB steps, then narrowing to 5 dB steps near the threshold. The ABR threshold for each frequency was determined by the lowest sound level that gave a measurable signal.

### Protein extraction and western blots

Mice were deeply anesthetized and sacrificed at P7. Mouse cochleae (*n* = 4 in each group) were carefully dissected from the temporal bone in ice-cold 0.01 M phosphate-buffered saline (PBS), and the total proteins of the membranous labyrinth were extracted using RIPA lysis buffer (P0013B, Beyotime Biotechnology, China). The protein concentrations were quantified using a bicinchoninic acid protein assay kit (P0012S, Beyotime Biotechnology, China). Equal amounts of protein samples were separated by electrophoresis on 12% sodium dodecyl sulfate–polyacrylamide gel electrophoresis gels and then transferred to polyvinylidenedifluoride (PVDF) membranes. The membranes were blocked in Tris-buffered saline (TBST with 0.1% Tween-20) containing 5% milk for 1 h. The Cx26 and β-actin proteins were detected using rabbit polyclonal antibodies against Cx26 (1:1,000 dilution, 512800, Invitrogen, USA) or rabbit polyclonal antibodies against β-actin (1:1000 dilution, 04-1116, Millipore, USA). After washing in TBST, the PVDF membranes were incubated with horseradish peroxidase (HRP)-conjugated goat anti-rabbit secondary antibody for 1 h at room temperature. Bands were visualized with an ECL reaction kit (P0018, Beyotime Biotechnology, China). The protein levels of Cx26 were measured by Quantity One 4.6.2 (Bio-Rad, USA) and normalized to the levels of β-actin in the corresponding lane.

### Cochlear tissue preparation and immunofluorescent labeling

For Cx26-null cell quantification, mice (*n* = 3) were sacrificed at P7. For cochlear HC and SC counting, mice (*n* = 4) were deeply anesthetized and sacrificed at P18 or P60. The cochleae were carefully dissected from the temporal bones and fixed in 4% paraformaldehyde in 0.01 M PBS for 1 h at room temperature. The flattened cochlear preparations were carefully dissected in ice-cold 0.01 M PBS. After blocking in a solution of 10% donkey serum with 1% Triton X-100 for 1 h, the samples were incubated with polyclonal rabbit anti-Cx26 antibodies (1:200 dilution, 512800, Invitrogen, USA), polyclonal rabbit anti-myosin7a antibodies (1:500 dilution, 25-6790, Proteus Bio-Sciences, USA), or polyclonal goat anti-sox2 antibodies (1:100 dilution, sc-17320, Santa Cruz Biotechnology, USA) diluted in 0.01 M PBS with 0.3% Triton X-100 overnight at 4°C. Samples were washed three times in 0.01 M PBS with 0.1% Tween-20 and then stained with Alexa Fluor 647 donkey anti-rabbit IgG or Alexa Fluor 594 donkey anti-goat IgG (1:200 dilution, ANT032 and ANT031, Antgene, China) for 1.5 h. 4',6-diamidono-2-phenylindole(DAPI,C1005, Beyotime Biotechnology, China) and phalloidin (0.05 mg/mL, P5282, Sigma, USA) were used for nucleus and F-actin staining, respectively. Images were taken with a laser scanning confocal microscope (Nikon, Japan). To better understand the spatial expression pattern of Cx26 in DCs, images from different levels of DCs were merged together.

### Resin sections and transmission electron microscopy

Mice (*n* = 3) were deeply anesthetized and sacrificed at P60. The cochleae were removed and fully fixed with a mixture of 2% paraformaldehyde and 2.5% glutaraldehydein 0.1 M PBS. The samples were decalcified for 48–72 h in 10% disodium EDTA (pH = 7.2) and post-fixed for 1 h in1% osmium tetroxide. After dehydration through a graded ethanol series, the samples were embedded in resin. The samples were sectioned (1.5 μm in thickness) and stained with toluidine blue (89640-5G, Sigma-Aldrich, USA) for light microscope observation. The ultrathin sections were stained with uranyl acetate and lead citrate for transmission electron microscopy examination (FEI Tecnai G2 20 TWIN, Thermo Fisher Scientific, USA).

### Scanning electron microscopy

Mice were sacrificed at P18, and the cochleae were removed and fully fixed with a mixture of 2% paraformaldehyde and 2.5% glutaraldehyde in 0.1 M PBS. After decalcification, the bony capsule and lateral walls were removed from the cochleae. The basal turn was exposed to show the morphology of the DC3s and OHC3s. Samples were subsequently dehydrated through a graded series of ethanol, critical point dried from liquid CO_2_ (HCP-2, Critical Point Dryer, HITACHI, Japan), mounted on stubs, and sputter-coated with a layer of gold (Eiko Engineering, Japan). Photographs were taken using a scanning electron microscope (VEGA 3 LMU, Tescan, Czechoslovakia).

### Statistical analysis

All data are presented as means ± s.e.m. and plotted with Sigma Plot (version 12.5, Systat Software, Inc., USA). The *t*-tests were performed in SPSS software (version 19, IBM SPSS Statistics, USA), and *P* < 0.05 was considered to be statistically significant.

## Supplementary information

FigureS1

FigureS2

supplementary figure legends
