# Supplementary figures and images for: The spatial distribution pattern of Connexin26 expression in supporting cells and its role in outer hair cell survival
Source: Cell Death Dis. 2018 Dec 5;9(12):1180. doi: 10.1038/s41419-018-1238-x (PMC6281596; doi:10.1038/s41419-018-1238-x)

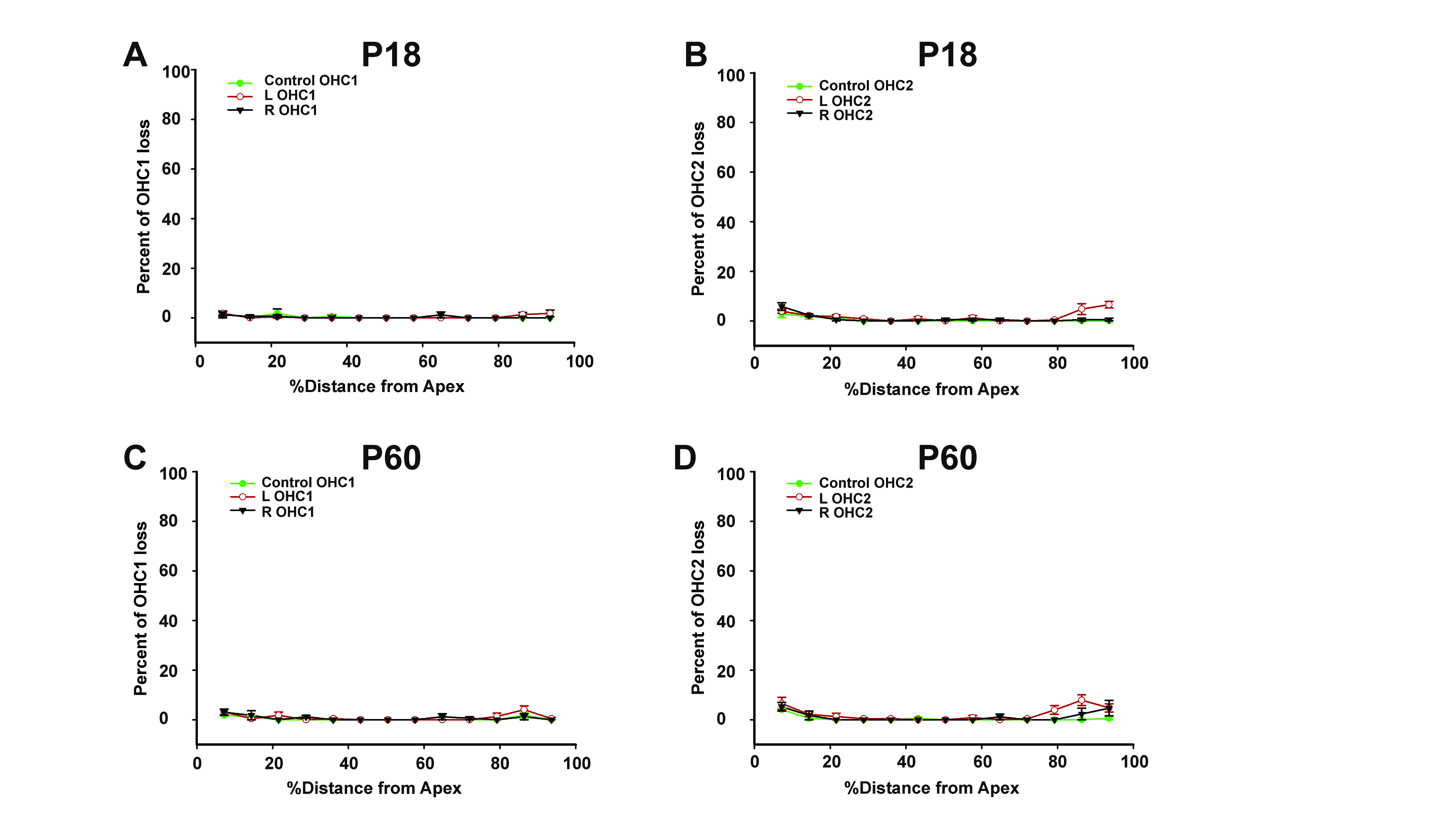

Supplement: Supplementary file 2 — FigureS2 [file 41419_2018_1238_MOESM2_ESM.tif]
