# Supplementary material for: The spatial distribution pattern of Connexin26 expression in supporting cells and its role in outer hair cell survival
Source: Cell Death Dis. 2018 Dec 5;9(12):1180. doi: 10.1038/s41419-018-1238-x (PMC6281596; doi:10.1038/s41419-018-1238-x)
Supplement: Supplementary file 3 — supplementary figure legends [file 41419_2018_1238_MOESM3_ESM.docx]

**Supplementary figure legends**

**Figure S1. Cx26 expression patterns in different types of supporting cells.** (A-C): Cx26 expression (red) in CCs from the control and experimental groups. (D-F): Cx26 expression in ISCs from the control and experimental groups. The scales in panel A represent 10 µm.

**Figure S2. OHC1 and OHC2 counts in the control and experimental groups.** (A and B): Quantifications of OHC1(A) or OHC2(B) loss at specific cochlear locations in the different groups at P18. (C and D): Quantifications of OHC1(C) or OHC2(D) loss at specific cochlear locations in the different groups at P60.
